# The Role of the Cytoskeleton and Myosin-Vc in the Targeting of KCa3.1 to the Basolateral Membrane of Polarized Epithelial Cells

**DOI:** 10.3389/fphys.2016.00639

**Published:** 2017-01-04

**Authors:** Rachel E. Farquhar, Ely Rodrigues, Kirk L. Hamilton

**Affiliations:** ^1^Department of Physiology, Otago School of Medical Sciences, University of OtagoDunedin, New Zealand; ^2^Department of Medicine, Otago School of Medical Sciences, University of OtagoDunedin, New Zealand

**Keywords:** K^+^ channels, targeting, myosin-Vc, microtubule, actin

## Abstract

Understanding the targeting of KCa3.1 to the basolateral membrane (BLM) of polarized epithelial cells is still emerging. Here, we examined the role of the cytoskeleton (microtubules and microfilaments) and Myosin-Vc (Myo-Vc) in the targeting of KCa3.1 in Fischer rat thyroid epithelial cells. We used a pharmacological approach with immunoblot (for the BLM expression of KCa3.1), Ussing chamber (functional BLM expression of KCa3.1) and siRNA experiments. The actin cytoskeleton inhibitors cytochalasin D (10 μM, 5 h) and latrunculin A (10 μM, 5 h) reduced the targeting of KCa3.1 to the BLM by 88 ± 4 and 70 ± 5%, respectively. Colchicine (10 μM, 5 h) a microtubule inhibitor reduced targeting of KCa3.1 to the BLM by 63 ± 7% and decreased 1-EBIO-stimulated KCa3.1 K^+^ current by 46 ± 18%, compared with control cells. ML9 (10 μM, 5 h), an inhibitor of myosin light chain kinase, decreased targeting of the channel by 83 ± 2% and reduced K^+^ current by 54 ± 8% compared to control cells. Inhibiting Myo-V with 2,3-butanedione monoxime (10 mM, 5 h) reduced targeting of the channel to the BLM by 58 ± 5% and decreased the stimulated current of KCa3.1 by 48 ± 12% compared with control cells. Finally, using siRNA for Myo-Vc, we demonstrated that knockdown of Myo-Vc reduced the BLM expression of KCa3.1 by 44 ± 7% and KCa3.1 K^+^ current by 1.04 ± 0.14 μA compared with control cells. These data suggest that the microtubule and microfilament cytoskeleton and Myo-Vc are critical for the targeting of KCa3.1.

## Introduction

The intermediate conductance, Ca^2+^-activated K^+^ channel (KCa3.1, KCNN4) is targeted to the basolateral membrane (BLM) in polarized epithelial cells where it aids in maintenance of the cellular potential required for normal cellular function (Devor et al., 1996; Ishii et al., 1997; Logsdon et al., 1997; Vandorpe et al., 1998). Thus, KCa3.1 plays critical roles in epithelial ion and fluid transport, cardiovascular and blood pressure regulation, endothelia function, and blood physiology (Grgic et al., 2009; Köhler et al., 2010; Wulff and Castle, 2010).

In the last 20 years, KCa3.1 has emerged as a key therapeutic target for a number of diseases and the modulation of this channel has been investigated in an attempt to develop therapeutic measures for ameliorating diseases such as autosomal dominant polycystic kidney disease (Albaqumi et al., 2008), vascular disease (Köhler, 2009; Damkjaer et al., 2012), and ulcerative colitis (Al-Hazza et al., 2012). Due to the recent discovery of mutations in KCa3.1 (Rapetti-Mauss et al., 2015) associated with an autosomal-dominant form of anemia (hereditary xerocytosis, Miller et al., 1971) the need to understand the role of KCa3.1 in disease had come under scrutiny.

Interestingly, in therapeutic development, many steps have been taken to regulate the K^+^ channel activity of KCa3.1 via pharmacological agonists or inhibitors (Wulff et al., 2001; Bradding and Wulff, 2009; Köhler et al., 2010; Yu et al., 2013), however, little research has been conducted to examine the mechanism of how KCa3.1 is targeted to the plasma membrane. Increased or decreased KCa3.1 activity can result in altered pathophysiological states (Albaqumi et al., 2008; Al-Hazza et al., 2012). Therefore, modulation of specific pathways in targeting KCa3.1 to the BLM may be a viable therapeutic approach.

Complex pathways ensure that apically and basolaterally destined proteins are correctly sorted and targeted to the appropriate membrane (Fölsch et al., 2009; Ang and Fölsch, 2012). As such, the cytoskeleton plays a significant role in the targeting of membrane proteins. The microtubule cytoskeleton is found throughout cells (Li et al., 2012) and it is composed of polarized, dynamic, and hollow tube shaped structures formed from tight helices of polymerized dimers of α and β tubulin (Mitchison and Kirschner, 1984). The formation of microtubules occurs via the polymerization of αβ-tubulin dimers that are controlled by the hydrolysis of β-GTP to β-GDP (Brouhard and Sept, 2012). The microtubule cytoskeleton has long been investigated as playing a role in protein targeting. Using pharmacological methods, the microtubule cytoskeleton has been implicated in the BLM targeting of proteins in epithelial cells including aquaporin 4 (AQP4; Mazzaferri et al., 2013) and V-ATPase (Brown et al., 1992).

Microfilaments (herein referred to as the actin cytoskeleton) are conserved across cells and are comprised of dynamic structures that play roles in trafficking, motility, stability, and muscle contraction (Hightower and Meagher, 1986; Semenova et al., 2008). Actin filaments (F-actin) consist of polymerized G-actin monomers. G-actin binds the existing F-actin strand via hydrolysis of G-(ATP)-actin monomers to G-(ADP)-actin (Frixione, 2000; Oda and Maéda, 2010). The actin cytoskeleton is associated with many accessory proteins, including Myosin Vc (Myo-Vc; Jacobs et al., 2009). Myosins function as actin motor proteins and bind actin filaments via cyclic phosphorylation and hydrolyzation, producing forces, and directional mechanical work in both muscle and non-muscle cells (De La Cruz et al., 1999). 35 classes of myosins have been distinguished on the basis of sequences of amino acids in their hydrolyzing domains (Odrontiz and Kollmar, 2007; Kneussel and Wagner, 2013), of these Myo-Vc was selected as a candidate gene. Myo-Vc is highly expressed in exocrine and epithelial tissues (Rodriguez and Cheney, 2002; Jacobs et al., 2009) and was chosen for this study, because, this motor protein is important in organelle and vesicle transport (Rosé et al., 2002; Desnos et al., 2007).

While Bertuccio et al. (2014) reported that Rab1 and Rab8 are important for the arrival of KCa3.1 to the BLM, little more investigation has been conducted to further explore the mechanism of the basolateral targeting of KCa3.1. In this study, we have investigated the role of the cytoskeleton and Myo-Vc in the basolateral targeting of KCa3.1 in a polarized epithelium. Here, we provide the first evidence that the targeting of KCa3.1 to the BLM is dependent on the microtubule and actin cytoskeleton, and specifically the actin motor protein Myo-Vc.

## Materials and methods

### Molecular biology

The biotin ligase acceptor peptide (BLAP) sequence (GLNDIFFEQKIEWHE) was inserted into the second extracellular loop of KCa3.1 as previously described (Balut et al., 2010a; Gao et al., 2010). KCa3.1-BLAP and BirA (biotin ligase) with an endoplasmic reticulum (ER) retention sequence, KDEL (BirA-KDEL; kindly provided by Dr. Alice Ting, Massachusetts Institute of Technology, Cambridge, MA, Chen et al., 2005; Howarth and Ting, 2008), were subcloned into a bicistronic plasmid, pBudCE4.1 (Invitrogen, ThermoFisher, Waltham, MA, USA) behind the EF-1α and CMV promoters, respectively (further details in Balut et al., 2010a,b; Gao et al., 2010; Balut et al., 2011).

### Cell culture and establishing a stably transfected FRT cell line

Fisher rat thyroid (FRT) cells were cultured in Nutrient Mixture F-12 media (Invitrogen) and supplemented with 10% fetal bovine serum and 1% penicillin-streptomycin (Life Technologies, ThermoFisher). The cells were cultured in a humidified 5% CO_2_/95% O_2_ incubator at 37°C. We established a stable cell line by transfecting the bicistronic plasmid pBudCE_4.1_ dually expressing KCa3.1-BLAP and BirA-KDEL with Lipofectamine 2000™ (Invitrogen) into FRT cells (FRT-KCa3.1-BLAP). Devor and coworkers (Gao et al., 2010) have previously demonstrated that insertion of the BLAP sequence into KCa3.1 did not affect the Ca^2+^ sensitivity, activation by DCEBIO, or the inhibition of the channel by clotrimazole. The stable cell line was maintained using zeocin (850 μg/ml, Life Technologies; protocol approved by the University of Otago Institutional Biological Safety Committee). Stably transfected FRT-KCa3.1-BLAP cells were seeded on to Transwell™ or Snapwell™ permeable supports (Corning Inc., Corning, NY) at a density of 5 × 10^5^ cells and cultured to form confluent epithelial monolayers (~3 days) in a humidified 5% CO_2_/95% O_2_ incubator at 37°C.

### Biotinylation and streptavidin labeling of KCa3.1-BLAP

Within the stable KCa3.1-BLAP—BirA-KDEL cell line, the BirA-KDEL is retained within the ER, therefore, once a KCa3.1-BLAP channel is assembled within the ER, the biotin ligase enzyme, Bir-A, biotinylates the channel prior to exiting of the channel from the ER, and being targeted to the plasma membrane. Once at the plasma membrane, streptavidin labeling of surface KCa3.1-BLAP was performed as previously described (Balut et al., 2010a; Bertuccio et al., 2014). Briefly, upon reaching confluence, cells were taken out of the incubator for labeling, all procedures and solutions were maintained at 4°C to prevent channel internalization. Cells were first washed with 2 ml of 4°C PBS with 1% bovine serum albumin (BSA) on both apical and basolateral sides of the permeable support filter to eliminate residual media. Since streptavidin is cell impermeable, the cells were labeled by applying streptavidin (10 μg/ml in PBS with 1% BSA) on the desired side (apical or basolateral, depending upon the experimental protocol) of the filter for 35 min at 4°C. After labeling, cells were washed three times with PBS with 1% BSA and three times with PBS to eliminate residue streptavidin and the cells were incubated for various periods of time at 37°C as indicated in the text (Balut et al., 2010a; Bertuccio et al., 2014).

### Reverse transfection

FRT-KCa3.1-BLAP cells were reverse transfected (5 pmol) with either the Myo-Vc siRNA plasmid (FRT-KCa3.1-BLAP+MyoVc-siRNA; Thermo Fischer Scientific) or the Universal Negative siRNA Control I plasmid (FRT-KCa3.1-BLAP+SC-siRNA; Thermo Fischer Scientific) for 48 h using Lipofectamine 2000™ (Invitrogen). FRT-KCa3.1-BLAP cells were seeded at a density of 7.5 × 10^4^ cells onto Transwell™ and Snapwell™ permeable support filters (Corning Inc.) and cultured to form a confluent epithelial monolayer in a humidified 5% CO_2_/95% O_2_ incubator at 37°C.

### Cytotoxicity tests

Cytotoxicity tests were conducted to determine the non-toxic concentration of the drugs for normal cell growth and integrity of FRT cells. Therefore, FRT-KCa3.1-BLAP cells were treated with cytochalasin D (Cyto D), latrunculin A (Lat A), ML9 [1-(5-Chloronaphthalene-1-sulfonyl)-1H-hexahydro-1,4-diazepine hydrochloride], or colchicine at 0, 5, 10, and 20 μM for 0, 3, and 5 h. Likewise to assess the toxicity of 2,3-butanedione monoxine (BDM), cells were treated with BDM at 0, 5, 10, and 20 mM for 0, 3, and 5 h. Normally, FRT-KCa3.1-BLAP cells were seeded at a density of 5 × 10^5^ cells and cultured to confluence on Transwell™ filters and counted post drug treatment with a haemocytometer at the designated incubation time point. There was only cytotoxic effects of FRT cells grown for the 5 h time point with 20 μM for Cyto D (Supplementary Figure 1A), Lat A (Supplementary Figure 1B), and colchicine (Supplementary Figure 1C) and no toxic effects on FRT cell growth treated with ML9 or BDM for any concentration or time point (data not shown). Therefore, 10 μM was used for examining the effect of Cyto D, Lat A, ML9, colchicine, and 10 mM BDM on the targeting of KCa3.1.

### Antibodies

For the detection of Myo-Vc, goat polyclonal anti-Myo-Vc [Myosin-Vc (Y-19): sc-160556] (1:1000) and HRP-linked polyclonal donkey anti-goat IgG (1:10,000) were purchased from Santa Cruz Biotechnology Incorporated (Santa Cruz, CA, USA). HRP-linked polyclonal donkey anti-rabbit IgG (1:2000) was purchased from Promega Corporation (Madison, WI, USA). To detect KCa3.1, rabbit polyclonal anti-streptavidin (1:1000) was obtained from GenScript (Piscataway, NJ, USA) and HRP-linked polyclonal donkey anti-rabbit IgG (1:2000) was purchased from Promega Corporation. For the detection of GAPDH, rabbit polyclonal anti-GAPDH (1:1000) was purchased from Sigma-Aldrich (St Louis, MO, USA).

### Immunoblot experiments

Cells were lysed and the protein was harvested as previously described (Balut et al., 2010b, 2011; Gao et al., 2010) and protein concentrations were determined by the BCA technique. 30 μg of protein was run per lane with a protein standard (BenchMark™, Invitrogen, Cat. No. 10748-010) added to a different lane and separated out on an 8% SDS-PAGE gel, and gels were run on a Hoefer Mighty Small II system (Cat. No. 80-6149-35, Amersham Biosciences Corp. Piscataway, NJ, USA) at 150 V for 90 min or until the dye front reached the bottom of the gel. After which, proteins were transferred onto a polyvinyl-diflouride membrane (Sigma, St. Louis, MO, USA) using the Trans-Blot® Turbo™ Transfer Starter System (Model 1704155, Bio-Rad, Hercules, CA, USA) in transfer buffer (25 mM Tris, 190 mM glycine, and 20% methanol) at 25 V for 30 min. Membranes were blocked overnight at 4°C in a TBS-T blocking solution (5% milk powder, 0.1% Tween 20). Membranes were then incubated in 1° Ab for 1 h at room temperature for the detection of either KCa3.1, Myo-Vc, or GAPDH (used as a loading control). Membranes were then washed extensively in TBS-T (0.1% Tween 20) and incubated in the appropriate 2° Ab for 1 h at room temperature. Membranes were then washed again in TBS-T (0.1% Tween 20) and detection was performed using West Pico Chemiluminescent Substrate (Roche Diagnostics, Indianapolis, IN, USA). Immunoblot band densities for KCa3.1 and Myo-Vc were normalized to the GAPDH loading control. All immunoblot data were quantified using Image J (NIH, vers. 1.51, Bethesda, MD, USA).

### Ussing chamber experiments

For conducting Ussing chamber experiments, FRT-KCa3.1-BLAP cells were cultured on Snapwell™ filters and grown to form a confluent monolayer. To facilitate a K^+^ gradient across the monolayer and promote transepithelial K^+^ transport, the apical (mucosal) chamber was filled with 5 ml of high K^+^ Ringer's (in mM) 145 potassium gluconate, 10 HEPES, 1 MgCl, 4 CaCl_2_, and 10 glucose (pH 7.4) and the basolateral (serosal) chamber was filled with a solution containing (in mM) 140 sodium gluconate, 5 potassium gluconate, 10 HEPES, 1 MgCl, 4 CaCl_2_ and 10 glucose (pH of 7.4). All solutions were maintained at 37°C. The CaCl_2_ was increased from the normal 1.2–4 mM to compensate for the Ca^2+^-buffering capacity of the gluconate anion (Durham, 1983). KCa3.1 activity was stimulated using KCa3.1 specific agonist 1-EBIO (100 μM, Devor et al., 1996) and inhibited by clotrimazole (10 μM, Devor et al., 1997). All measurements were recorded by a VCC MC Ussing chamber system (Physiologic Instruments, San Diego, CA, USA). The Ussing chamber was comprised of an Easymount chamber system with 8-chamber voltage and current units (Physiologic Instruments). Prior to mounting a filter in a chamber, the Ussing chamber was zeroed to remove any offsets (Clarke, 2009). The monolayer was considered to have sufficient integrity when the FRT monolayer exhibited a *R* ≥ 500 Ω; therefore experiments where *R* did not achieve ≤500 Ω were not used for data analyses. Wild type FRT cells (WT) served as controls for the Ussing experiments as seen for the colchicine (**Figure 4C**) and ML9 (**Figure 5C**) experiments. The slight changes in the current traces for the WT cells with the addition of 1-EBIO and clotrimazole were due to the vehicle (ethanol) that was verified in vehicle control experiments (data not shown).

Ussing chamber experiments were conducted to demonstrate the specificity of clotrimazole for inhibiting 1-EBIO-stimulated K^+^ current of KCa3.1 in our FRT-KCa3.1-BLAP stable cell line. As can be seen in Supplementary Figure 2, 1-EBIO (100 μM) increased K^+^ current which was blocked by clotrimazole (10 μM). The remaining basal current was blocked by barium (10 mM).

### Chemicals

All chemicals were purchased from Sigma-Aldrich, unless otherwise stated. DMSO was used as a vehicle for Cyto D, Lat A, ML9, and BDM. The vehicle for colchicine, 1-EBIO, and clotrimazole was ethanol.

### Statistical analyses

In this study “n” is indicative of the number of experiment repeats for different passages of cells; *P* ≤ 0.05 was considered statistically significant and all data are presented as mean ± SEM. Cytotoxic tests were analyzed using the parametric one way analysis of variance (one way ANOVA) followed by a Bonferroni post-test. Recorded Ussing traces were analyzed using Microsoft Excel (2010) and GraphPad Prism 5 (GraphPad Software, Inc., La Jolla, CA). A non-parametric Kruskal-Wallis with a Dunn's post-test was used to compare traces of 1-EBIO stimulated KCa3.1 currents of Ussing chamber data were normalized to FRT-KCa3.1-BLAP controls. To compare the normalized values of the immunoblot band intensities, statistical analysis was performed using the non-parametric Kruskal-Wallis test followed by a with Dunn's post-test. A one-way ANOVA followed by a Bonferroni post-test was used to compare trace peaks of KCa3.1 current in FRT-KCa3.1-BLAP, FRT-KCa3.1-BLAP+SC-siRNA, and FRT-KCa3.1-BLAP+MyoVc-siRNA cell lines.

## Results

### Localization of KCa3.1 in polarized FRT cells

**Figure 1:**
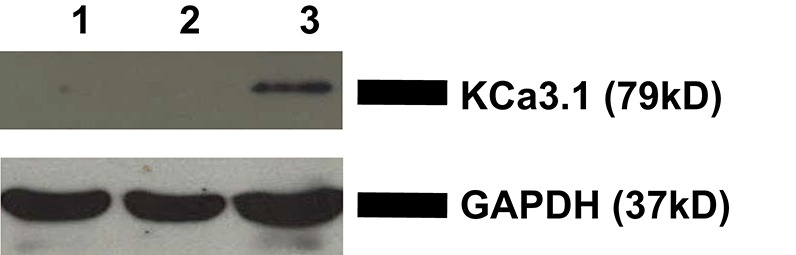
**Localization of KCa3.1 in stably transfected FRT cell line**. FRT-KCa3.1-BLAP cells were grown to confluence on Transwell™ filters. Lane 1: Negative control—non-labeled FRT-KCa3.1-BLAP cells. Lane 2: Apically streptavidin labeled FRT-KCa3.1-BLAP cells of the stably FRT cell line. Lane 3: Basolaterally-labeled FRT-KCa3.1-BLAP cells of the stably FRT cell line. GAPDH was used as a loading control. This sidedness experiment confirms that KCa3.1 is trafficked to the basolateral membrane of the stably transfected FRT-cell line. Thirty micrograms of protein was loaded per lane (*n* = 4).

To verify the membrane localization of KCa3.1, FRT-KCa3.1-BLAP cells were cultured on Transwell™ filters and labeled with streptavidin at either the apical or basolateral membrane. This was followed by immunoblot blot experiments using streptavidin and GAPDH antibodies as described in the Section Materials and Methods. As seen in Figure 1, KCa3.1 is expressed at the basolateral membrane of polarized FRT-KCa3.1-BLAP cells with no expression of the channel at the apical membrane (*n* = 4). These results confirm what we have previously reported using the FRT-KCa3.1-BLAP cell line (Bertuccio et al., 2014).

### The role of the microfilament (actin) cytoskeleton in the BLM targeting of KCa3.1

**Figure 2:**
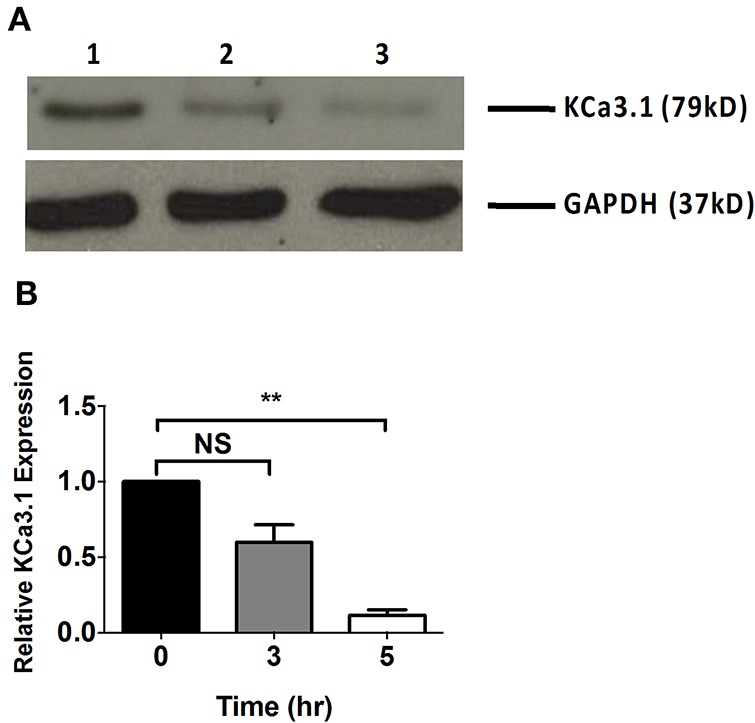
**The effect of cytochalasin D (Cyto D) on the targeting of KCa3.1 to the BLM of FRT cells. (A)** Immunoblot of KCa3.1 expression in response to exposure to Cyto D: FRT-KCa3.1-BLAP cells were incubated with Cyto D (10 μM) for 0, 3, and 5 h. Lane 1: untreated FRT-KCa3.1-BLAP cells, *t* = 0 h. Lane 2: FRT-KCa3.1-BLAP cells + Cyto D, *t* = 3 h. Lane 3: FRT-KCa3.1-BLAP cells + Cyto D, *t* = 5 h. GAPDH was used as a loading control. **(B)** Immunoblot quantification indicated that Cyto D reduced KCa3.1 expression at the BLM by 88 ± 4% at *t* = 5 h with respect to *t* = 0 h, (*n* = 5, ^**^P ≤ 0.01). NS, not significant.

**Figure 3:**
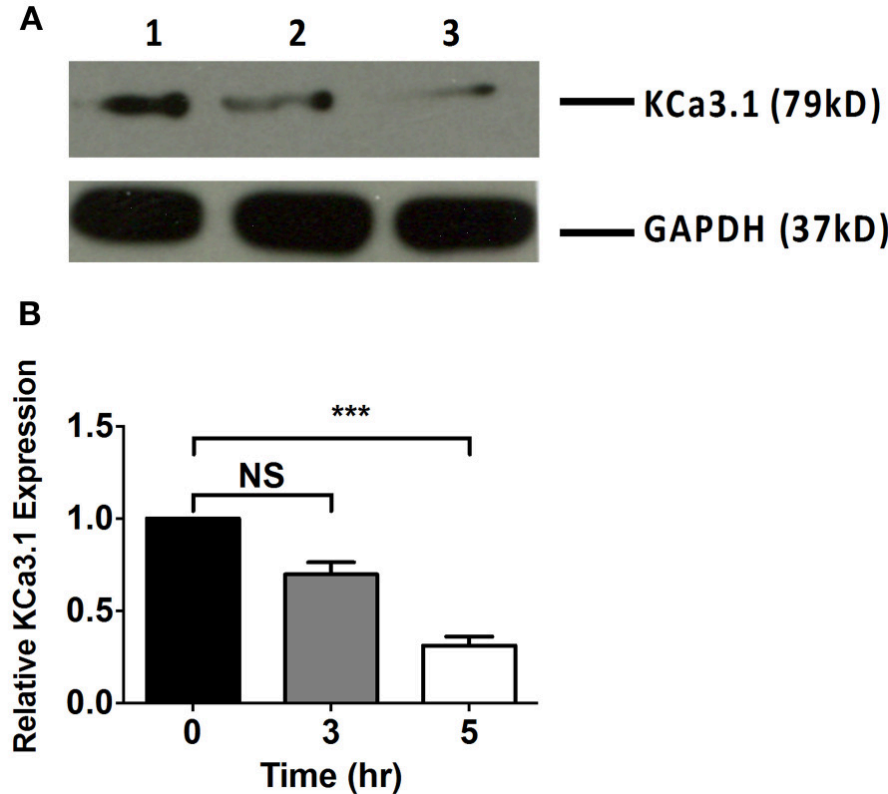
**The effect of latrunculin A (Lat A) on the targeting of KCa3.1 to the BLM of FRT cells. (A)** Immunoblot of KCa3.1 expression in response to exposure to Lat A: FRT-KCa3.1-BLAP cells were incubated with Lat A (10 μM) for 0, 3, and 5 h. Lane 1: untreated FRT-KCa3.1-BLAP, *t* = 0 h. Lane 2: FRT-KCa3.1-BLAP+ Lat A, *t* = 3 h. Lane 3: FRT-KCa3.1-BLAP+ Lat A, *t* = 5 h. GAPDH was used as a loading control. **(B)** Immunoblot quantification demonstrated that Lat A reduced KCa3.1 expression at the BLM by 70 ± 5% at *t* = 5 h with respect to *t* = 0 h, (*n* = 5, ^***^P ≤ 0.001). NS, not significant.

Microfilaments are comprised of actin and play an important role in intracellular trafficking of proteins and trafficking proteins by exocytosis and endocytosis (Conner and Schmid, 2003; Lee et al., 2008). Inhibitors of actin can modify polymerization of either F-actin or G-actin cytoskeleton, thus, reducing protein transport (Casella et al., 1981; Yarmola et al., 2000). We hypothesized that interrupting actin formation would result in reduced targeting of KCa3.1 to the BLM. Therefore, we tested whether Cyto D that inhibits F-actin and Lat A that inhibits G-actin (Casella et al., 1981; Yarmola et al., 2000) alter targeting of KCa3.1 to the BLM. Therefore, immunoblot experiments were conducted as described above. FRT-KCa3.1-BLAP cells were treated with either Cyto D or Lat A at 10 μM for 0, 3, and 5 h. As can be seen in Figure 2, FRT-KCa3.1-BLAP cells treated with Cyto D for 5 h exhibited a reduced BLM expression of KCa3.1 by 88 ± 4% (*P* ≤ 0.01) compared to untreated control cells (*t* = 0 h, *n* = 5). A similar effect was observed in FRT-KCa3.1-BLAP cells treated with Lat A (Figure 3) where there was a reduced BLM expression of KCa3.1 by 70 ± 5% (*P* ≤ 0.001) compared to untreated control cells (*t* = 0 h, *n* = 5). These data demonstrate, for the first time, that the targeting of KCa3.1 to the BLM of a polarized epithelium is dependent upon the intact actin cytoskeleton.

### The role of the microtubule cytoskeleton in the targeting of KCa3.1

**Figure 4:**
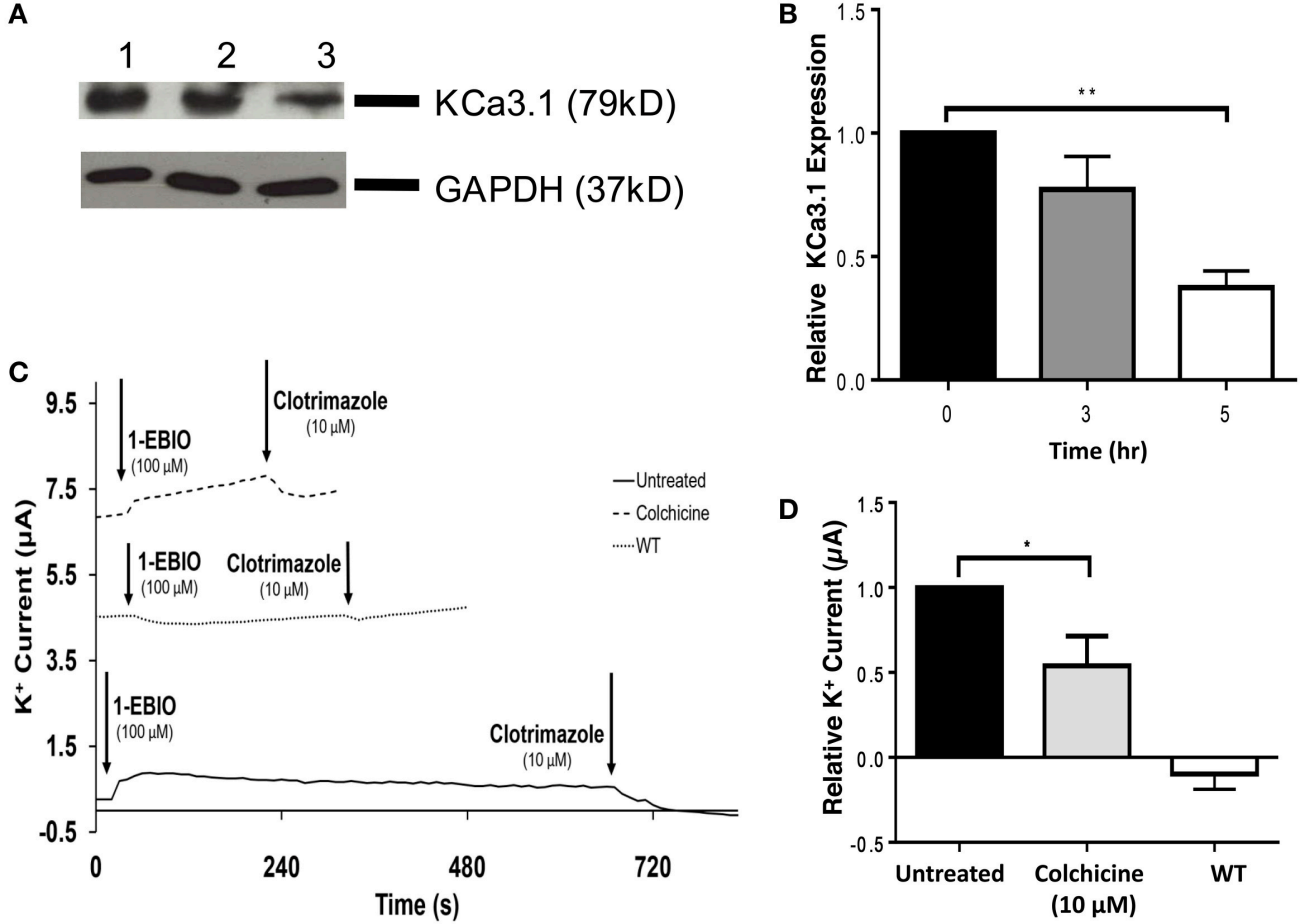
**The effect of colchicine on the BLM expression and function of KCa3.1. (A)** Basolateral streptavidin was used to label KCa3.1 and incubated with colchicine (10 μM) for 0, 3, or 5 h. Thirty micrograms of protein was run per lane. Lane 1: Untreated FRT-KCa3.1-BLAP, *t* = 0 h. Lane 2: FRT-KCa3.1-BLAP+colchicine, *t* = 3 h. Lane 3: FRT-KCa3.1-BLAP+colchicine, *t* = 5 h. GAPDH was used as a loading control. **(B)** Immunoblot quantification, at *t* = 5 h, colchicine decreased KCa3.1 expression in FRT-KCa3.1-BLAP cells by 63 ± 7% relative *t* = 0 h (*n* = 5, ^**^P ≤ 0.01). **(C)** A representative Ussing chamber trace of FRT-KCa3.1-BLAP cells treated with colchicine (10 μM). KCa3.1 specific agonist 1-EBIO (100 μM) was applied once a stable baseline current was achieved resulting in an increased K^+^ current. K^+^ current was inhibited by the addition of clotrimazole (10 μM). 1-EBIO elicited a significantly reduced K^+^ current in FRT-KCa3.1-BLAP cells treated with colchicine (dashed line) compared to untreated FRT-KCa3.1-BLAP cells (solid line). This current was completely blocked by clotrimazole (10 μM). WT FRT cells (WT, dotted line) served as controls and these cells exhibited no effect of 1-EBIO or clotrimazole. The minute change in current for WT was due to the vehicle based on vehicle control experiments (data not shown). **(D)** Bar graph of K^+^ current peak of FRT-KCa3.1-BLAP cells and FRT-KCa3.1-BLAP+colchicine cells treated with 1-EBIO. K^+^ current are normaiized to FRT-KCa3.1-BLAP cells. Colchicine decreased the 1-EBIO stimulated current by 46 ± 18% relative to FRT-KCa3.1-BLAP cells. (*n* = 6, ^*^P ≤ 0.05).

The role of the microtubule cytoskeleton has been documented in the trafficking of basolateral membrane proteins such as AQP4 and the V-ATPase (Brown et al., 1992; Mazzaferri et al., 2013) with using colchicine as an inhibitor of microtubule function. Therefore, we examined the effects of colchicine on confluent FRT-KCa3.1-BLAP cells to determine whether the microtubule cytoskeleton is crucial in the targeting of KCa3.1 to the BLM. The functional expression of KCa3.1 at the BLM was examined with immunoblot and Ussing chamber experiments. Therefore, FRT-KCa3.1-BLAP cells were seeded on to filters and incubated with colchicine (10 μM) for 0, 3, and 5 h. As demonstrated by immunoblot experiments (Figures 4A,B), at 5 h, the expression of KCa3.1 at the BLM was reduced by 63 ± 7% (*P* ≤ 0.01, *n* = 5) with respect to cells not treated with colchicine. To examine the effects of colchicine on the functional expression of FRT-KCa3.1-BLAP at the BLM, results from Ussing chamber experiments demonstrated that FRT-KCa3.1-BLAP cells treated with colchicine (for 5 h) had reduced K^+^ current (46 ± 18%, *P* ≤ 0.05, *n* = 6) compared to untreated FRT-KCa3.1-BLAP cells (Figures 4C,D, *n* = 6).

Collectively, these data provide the first experimental evidence that colchicine, via potential disruption of microtubule structure, reduced the BLM and functional expression of KCa3.1 of polarized epithelial cells. As such, these data suggest that an intact microtubule cytoskeleton is crucial for the proper targeting of KCa3.1 to the BLM.

### The role of light chain regulated myosin in the targeting of KCa3.1

**Figure 5:**
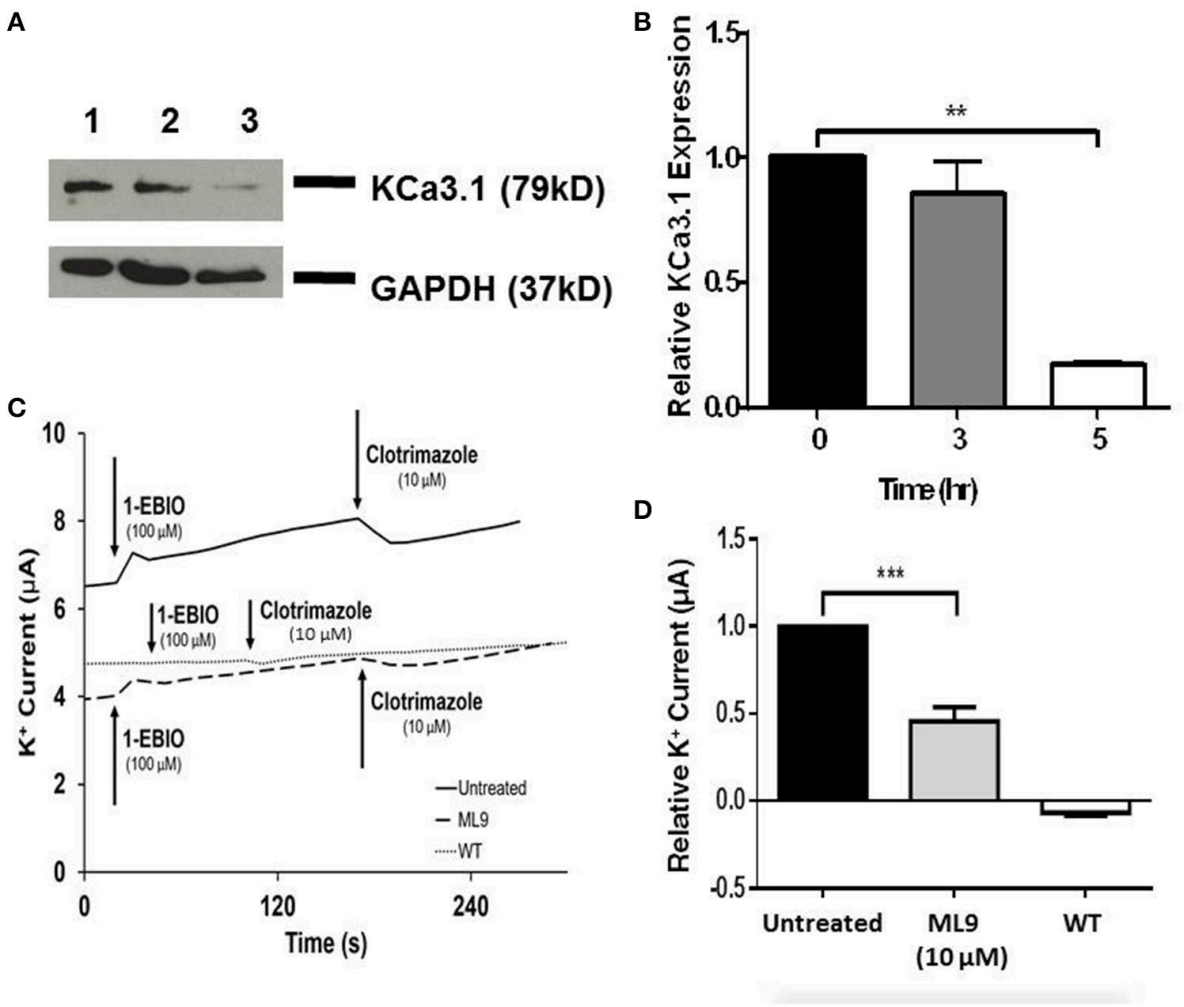
**The effect of ML9 on the BLM expression and function of KCa3.1 in response to the inhibition of light chain kinase regulated myosins. (A)** Immunoblot of KCa3.1 expression in response to exposure to ML9. FRT-KCa3.1-BLAP cells were incubated with ML9 (10 μM) for 0, 3, or 5 h. Lane 1: untreated FRT-KCa3.1-BLAP cells, *t* = 0 h. Lane 2: FRT-KCa3.1-BLAP+ML9, *t* = 3 h. Lane 3: FRT-KCa3.1-BLAP+ML9, *t* = 5 h. GAPDH was used as a loading control. **(B)** Immunoblot quantification. ML9 reduced KCa3.1 expression at the membrane by 83 ± 2% *t* = 5 h with respect to *t* = 0 h, (*n* = 5, ^**^P ≤ 0.01). **(C)** Ussing chamber experiment with FRT-KCa3.1-BLAP cells with 5 h treatment of ML9 (10 μM). A standard trace is shown of ML9 treated and untreated FRT-KCa3.1-BLAP cells. 1-EBIO elicited a significantly reduced K^+^ current in FRT-KCa3.1-BLAP cells treated with ML9 (dashed line) compared to untreated FRT-KCa3.1-BLAP cells (solid line). This current was completely blocked by clotrimazole (10 μM). WT FRT cells (WT, dotted line) served as controls and these cells exhibited no effect of 1-EBIO or clotrimazole. The minute change in current for WT was due to the vehicle based on vehicle control experiments (data not shown). **(D)** Bar graph of K^+^ current of FRT-KCa3.1-BLAP cells and FRT-KCa3.1-BLAP+ML9 cells were treated with 1-EBIO. K^+^ current is normalized to FRT-KCa3.1-BLAP cells. ML9 decreased the 1-EBIO stimulated current by 54 ± 8% relative to FRT-KCa3.1-BLAP cells (*n* = 7, ^***^P ≤ 0.001).

Since, we have established that the actin cytoskeleton is crucial in the targeting of KCa3.1, our focus shifted to the potential role of myosins in the targeting of KCa3.1. If a myosin is critical in this process then our initial step was to determine whether a myosin light chain kinase (MLCK) was involved in the targeting of KCa3.1. This was investigated by using ML9, a MLCK inhibitor (Ishikawa et al., 1988). Therefore, the role of light chain regulated myosins in the targeting of KCa3.1 was investigated by administering ML9 (10 μM) for 0, 3, and 5 h to confluent FRT-KCa3.1-BLAP cells. Cells were seeded on to filters for immunoblot and Ussing chamber experiments were conducted as described above. As seen in Figures 5A,B, after exposure to ML9 for 5 h, the BLM expression of KCa3.1 was significantly reduced by 83 ± 2% (*P* ≤ 0.01) compared to cells not incubated with ML9 (*t* = 0 h, *n* = 5). To determine the effects of ML9 on the functional expression of KCa3.1, results from Ussing chamber experiments (Figures 5C,D) demonstrated that the inhibition of light chain kinase regulated myosins by ML9 reduced K^+^ current of KCa3.1 by 54 ± 8% (*P* ≤ 0.001) compared to control FRT-KCa3.1-BLAP cells (*t* = 0 h, *n* = 7). The results demonstrate, for the first time, that the targeting of KCa3.1 to the BLM of an epithelium is a light chain regulated myosin-dependent mechanism.

### The role of Myosin-Vc in the targeting of KCa3.1 to the BLM

**Figure 6:**
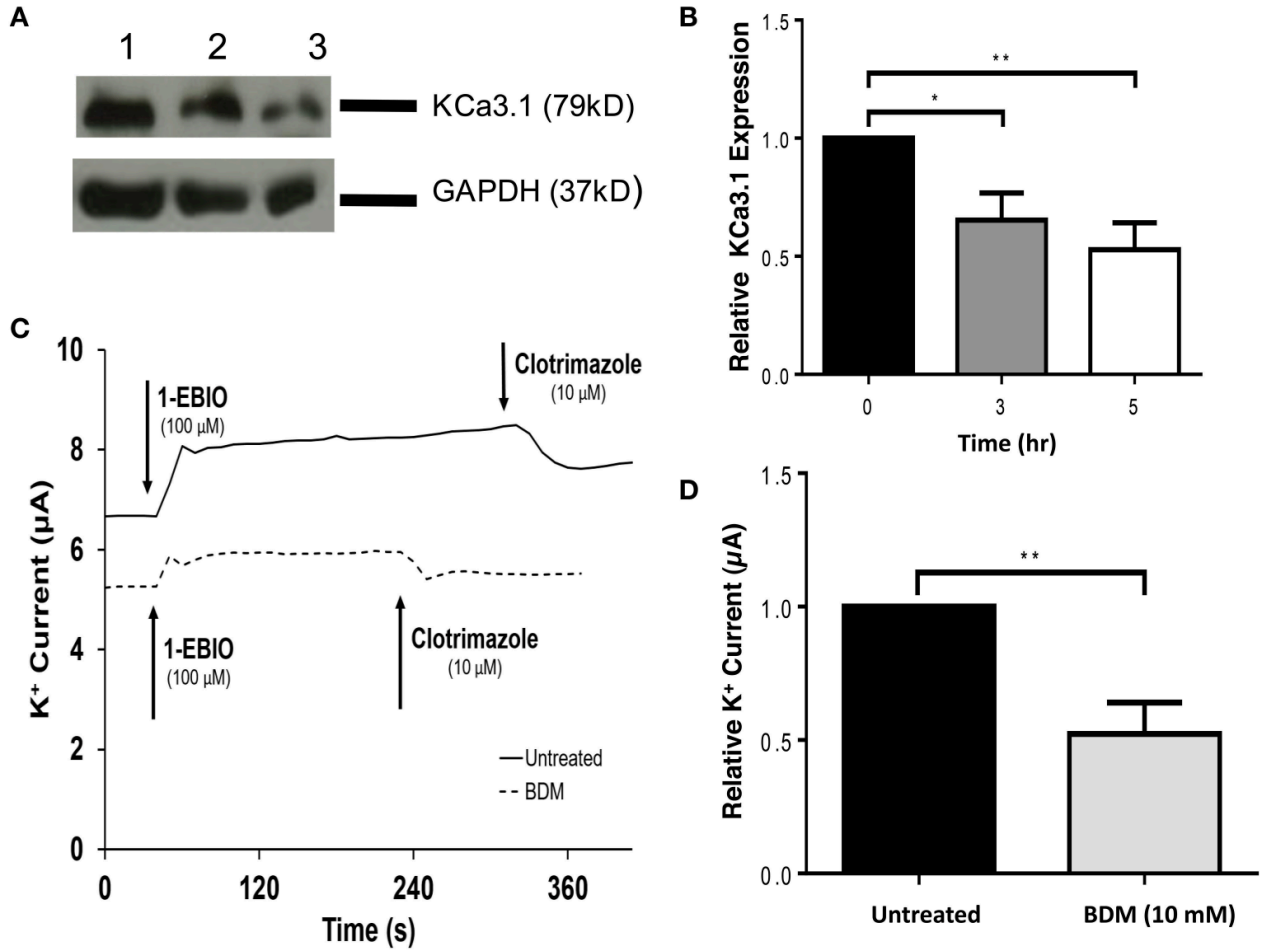
**The effect of BDM on the BLM expression and function of KCa3.1 in response to the inhibition of light chain kinase regulated myosins. (A)** Immunoblot of KCa3.1 expression in response to exposure to BDM. FRT-KCa3.1-BLAP cells are incubated with BDM (10 mM) for 0, 3, and 5 h. Lanes were loaded with 30 μg of protein. Lane 1: FRT-KCa3.1-BLAP cells, *t* = 0 h. Lane 2: FRT-KCa3.1-BLAP+BDM, *t* = 3 h. Lane 3: FRT-KCa3.1-BLAP+BDM, *t* = 5 h. GAPDH was used as a loading control. **(B)** Immunoblot quantification. At *t* = 5 h, BDM reduced KCa3.1 expression at the membrane by 58 ± 5% with respect to *t* = 0 h (*n* = 5, ^**^P ≤ 0.01). **(C)** Ussing chamber experiment of FRT-KCa3.1-BLAP cells with 5 h BDM treatment. A standard trace is shown of FRT-KCa3.1-BLAP cells and FRT-KCa3.1-BLAP cells treated with 10 mM BDM for *t* = 5 h. 1-EBIO generated a significantly reduced K^+^ current in FRT-KCa3.1-BLAP cells treated with BDM (dashed line) compared to untreated FRT-KCa3.1-BLAP cells (solid line). This current was completely blocked by clotrimazole (10 μM). WT FRT cells (WT, dotted line) served as controls and these cells exhibited no effect of 1-EBIO or clotrimazole (trace not shown due to clarity). **(D)** Bar graph of K^+^ current of FRT-KCa3.1-BLAP and FRT-KCa3.1-BLAP+BDM cells treated with 1-EBIO. K^+^ current is normalized to data for FRT-KCa3.1-BLAP cells. BDM decreased the 1-EBIO stimulated current by 48 ± 12% relative to FRT-KCa3.1-BLAP cells. (^*^P < 0.05, *n* = 5) (^**^P ≤ 0.01, *n* = 8).

Having established a role of MLCK regulated myosins in the targeting of KCa3.1, we next examined a myosin that might participate in the BLM targeting of KCa3.1. Indeed, Myo-Vc was selected as a possible motor protein as it is highly expressed in epithelial tissues (Rodriguez and Cheney, 2002; Jacobs et al., 2009). To carry out these experiments, 2,3-butanedione monoxime (BDM) was chosen as an inhibitor of Myo-V (Uemura et al., 2004). As above, the effects of BDM on the targeting of KCa3.1 expression and membrane function were examined as described above. The effect of the inhibition of Myo-V on the expression of KCa3.1 was investigated by treating FRT-KCa3.1-BLAP cells with BDM (10 mM) for 0, 3, and 5 h. After 5 h exposure to BDM, the BLM expression of KCa3.1 was reduced by 58 ± 5% (*P* ≤ 0.01) compared with untreated FRT-KCa3.1-BLAP cells (Figures 6A,B, *t* = 0 h, *n* = 5). Similarly, results from Ussing chamber experiments (Figures 6C,D) demonstrated that BDM reduced the current of FRT-KCa3.1-BLAP cells by 48 ± 12% (*P* ≤ 0.01) compared with non-treated FRT-KCa3.1-BLAP cells (*t* = 0 h, *n* = 8). These results suggest that Myo-V play a role in the targeting of KCa3.1 to the BLM of epithelial cells.

**Figure 7:**
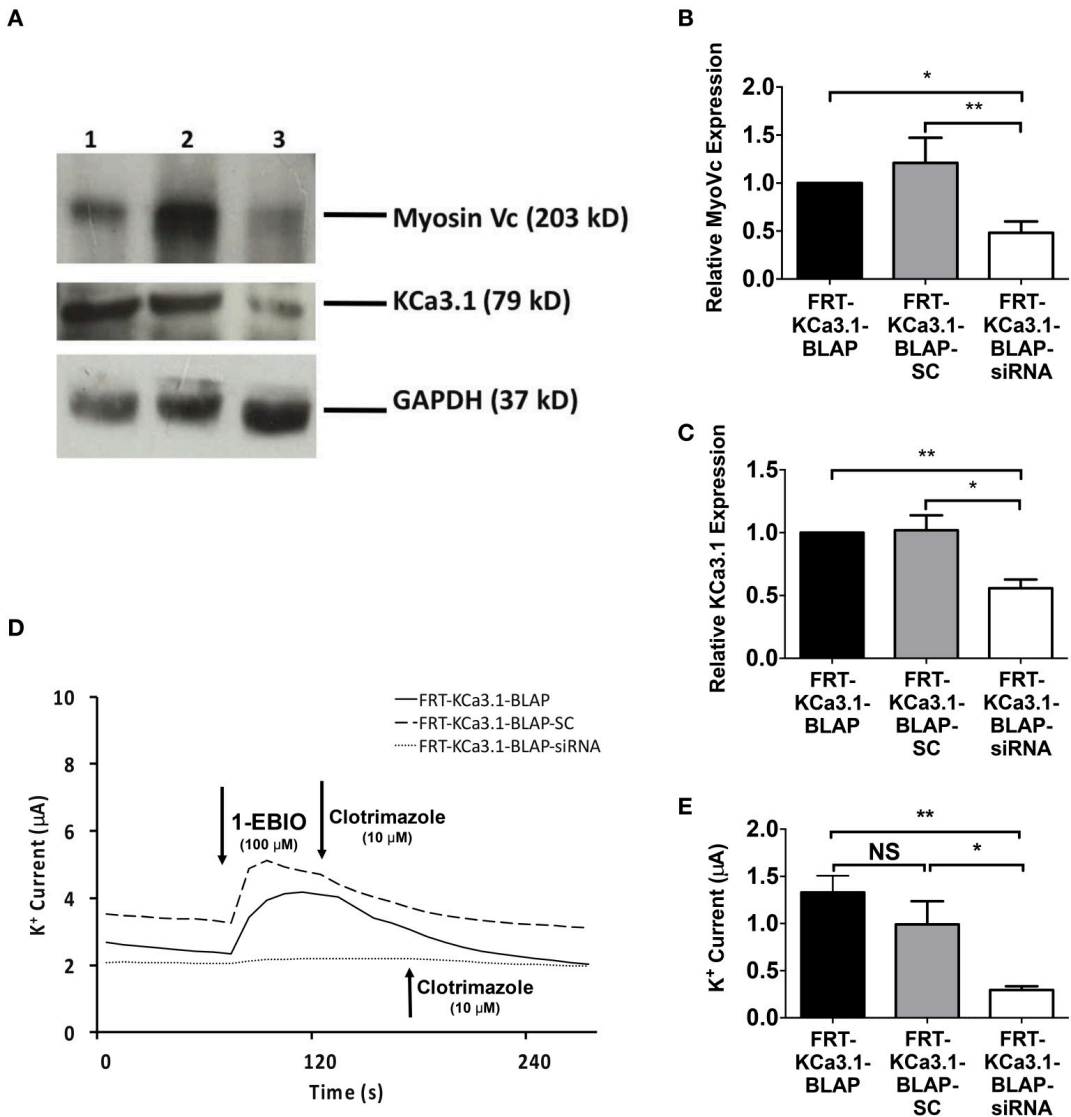
**The membrane expression and function of KCa3.1 in response to the siRNA knockdown of Myosin-Vc**. FRT-KCa3.1-BLAP were reverse transfected (5 pmol) with a scrambled siRNA (FRT-KCa3.1-BLAP-SC) or siRNA to knock down MyoVc (FRT-KCa3.1-BLAP-siRNA). **(A)** FRT-KCa3.1-BLAP-SC, and FRT-KCa3.1-BLAP-siRNA cells were run on an immunoblot. Lanes were loaded with 30 μg of protein. MyoVc and KCa3.1 were detected in FRT-KCa3.1-BLAP cells. Lane 1: FRT-KCa3.1-BLAP, Lane 2: FRT-KCa3.1-BLAP-SC, and Lane 3: FRT-KCa3.1-BLAP-siRNA. GAPDH was used as a loading control. **(B)** MyoVc expression in FRT-KCa3.1-BLAP-siRNA cells was reduced by 52 ± 11% (*n* = 6, ^*^P ≤ 0.05) with respect to FRT-KCa3.1-BLAP cells. **(C)** In the presence of knockdown of Myo-Vc, the KCa3.1 membrane expression in FRT-KCa3.1-BLAP-siRNA cells was reduced by 44 ± 7% (*P* ≤ 0.01) with respect to FRT-KCa3.1-BLAP cells. **(D)** Ussing chamber experiment of FRT-KCa3.1-BLAP cells with Myosin-Vc knock down. Representative traces of FRT-KCa3.1-BLAP cells (solid line), FRT-KCa3.1-BLAP-SC (dashed line) and FRT-KCa3.1-siRNA cells (dotted line) are shown. 1-EBIO promoted a significantly reduced K^+^ current in the FRT-KCa3.1-BLAP-siRNA cells compared to FRT-KCa3.1-BLAP cells or the FRT-KCa3.1-BLAP-SC. This current was abated by clotrimazole (10 μM). **(E)** Bar graph of K^+^ current of FRT-KCa3.1-BLAP, FRT-KCa3.1-BLAP-SC, and FRT-KCa3.1-siRNA cells were treated with 1-EBIO. FRT-KCa3.1-BLAP-SC cells did not exhibit K^+^ current different relative to FRT-KCa3.1-BLAP cells (*n* = 6, *P* ≥ 0.05). FRT-KCa3.1-BLAP-siRNA cells demonstrated a current significantly reduced by 1.04 ± 0.14 μA (^*^P ≤ 0.01) with respect to FRT-KCa3.1-BLAP cells, and a K^+^ current significantly reduced by 0.7 ± 0.2 μA (^*^P ≤ 0.05) with respect to FRT-KCa3.1-BLAP-SC cells was also recorded. (*n* = 6, ^**^P < 0.01) (*n* = 6, ^*^P ≤ 0.05).

In order to determine, indeed, that Myo-Vc plays a role in the targeting of KCa3.1, Myo-Vc was targeted using a siRNA approach. Therefore, FRT-KCa3.1-BLAP cells were transfected with either a universal negative control scrambled control siRNA (FRT-KCa3.1-BLAP-SC) or Myo-Vc specific siRNA (FRT-KCa3.1-BLAP-siRNA). Immunoblot data suggest that FRT-KCa3.1-BLAP cells transfected with a Myo-Vc-siRNA had a reduced cellular expression of Myo-Vc and membrane expression of KCa3.1. Relative to control FRT-KCa3.1-BLAP cells, the expression of Myo-Vc was reduced by 52 ± 11% (Figure 7A, upper panel, and Figure 7B, *P* ≤ 0.05, *n* = 6) and membrane expression of KCa3.1 was reduced by 44 ± 7% (Figure 7A, middle panel, and Figure 7C, *P* ≤ 0.01, *n* = 6) in FRT-KCa3.1-BLAP-siRNA cells, respectively. Relative to FRT-KCa3.1-BLAP-SC cells, the expression of Myo-Vc and KCa3.1 was reduced by 73 ± 15% (Figure 7B, *P* ≤ 0.05, *n* = 6) and 46 ± 5% (Figure 7C, *P* ≤ 0.05, *n* = 6) in FRT-KCa3.1-BLAP-siRNA cells, respectively. Results from the Ussing chamber experiments corroborated the immunoblot data in that in the presence of knockdown of Myo-Vc, the K^+^ current of FTR-KCa3.1-BLAP-siRNA cells was reduced by 1.04 ± 0.14 μA (dotted line, *P* ≤ 0.01, *n* = 6) with respect to FRT-KCa3.1-BLAP cells (Figure 7D, solid line, and Figure 7E). Relative to FRT-KCa3.1-BLAP-SC cells (Figure 7D, dashed line), KCa3.1 specific K^+^ current was reduced by 0.7 ± 0.2 μA (*P* ≤ 0.05, *n* = 6) in FRT-KCa3.1-BLAP-siRNA cells compared to control SC cells (Figures 7D,E).

These results are the first to suggest that the targeting of KCa3.1 to the BLM of epithelial cells is a Myo-Vc-dependent process.

## Discussion

Our understanding of the targeting of KCa3.1 is still emerging. Recently, Devor and colleagues made an important contribution to this field by reporting that trafficking of KCa3.1 is a Rab1- and Rab 8-dependent process (Bertuccio et al., 2014). Our results advance the field by providing the first experimental evidence that the microtubule and microfilament cytoskeleton and Myo-Vc are critical for the targeting of KCa3.1 to the BLM of epithelial cells.

The microtubules are considered an important component of epithelial cells and colchicine has been employed to interrupt the microtubule cytoskeleton in studying the basolateral trafficking of proteins in epithelial cells (Brown et al., 1992). Therefore, colchicine was used to examine the role of the microtubule function in the targeting of KCa3.1 to the BLM. Indeed, colchicine reduced the functional expression of KCa3.1 at the BLM of FRT-KCa3.1-BLAP cells (Figure 4). The reduced basolateral delivery of KCa3.1 suggests that, similar to trafficking of AQP4, the microtubule cytoskeleton is required for targeting of the KCa3.1 (Mazzaferri et al., 2013). Interestingly, others have described that the basolateral trafficking of Na^+^/K^+^-ATPase (Boll et al., 1991) and A2R receptors (Saunders and Limbird, 2000) are microtubule independent. This may suggest that certain basolateral targeting pathways are microtubule-dependent while others are not. It should also be noted that while we have demonstrated a reduced BLM expression of KCa3.1 in cells with compromised microtubule cytoskeleton, we are not aware if KCa3.1 is mis-targeted to the apical membrane. Future experiments investigating the potential apical mislocalization of KCa3.1 with sidedness experiments (similar to Figure 1) would address whether KCa3.1 is mis-targeted to the apical membrane or retained within the cell. Nevertheless, the focus of this study was on the basolateral targeting of KCa3.1, as such, those experiments were outside the scope of the current study.

### The role of Myosin-Vc in the targeting of KCa3.1

It was important to determine if the actin cytoskeleton was involved in the basolateral targeting of KCa3.1. Therefore, experiments were conducted by depolymerizing the actin cytoskeleton by administering Cyto D or Lat A to FRT-KCa3.1-BLAP cells and examining the membrane expression of KCa3.1. Immunoblot data demonstrated that FRT-KCa3.1-BLAP cells, with compromised actin cytoskeleton, had a reduced expression of KCa3.1 at the BLM relative to cells with an intact actin cytoskeleton (Figures 2, 3). Following this, we examined the role of actin motor proteins in the targeting of KCa3.1. We selected ML9 to inhibit myosins containing light chains that were regulated by MLCK (Ishikawa et al., 1988). Using immunoblot and Ussing chamber experiments, we determined that when FRT-KCa3.1-BLAP cells were exposed to ML9 there was a reduced membrane and functional expression of KCa3.1 at the BLM (Figure 5). These data demonstrate that the targeting of KCa3.1 to the BLM of polarized epithelial cells is dependent upon MLCK regulated myosins.

There are 35 classes of myosins of which 13 are present in humans and many of these myosins are MLCK regulated (Odrontiz and Kollmar, 2007; Kneussel and Wagner, 2013). One class of MLCK-regulated myosin is Myo-V; which is highly expressed in epithelial tissues and involved in targeting of organelles and vesicles containing cargo (Rodriguez and Cheney, 2002; Rosé et al., 2002; Desnos et al., 2007; Jacobs et al., 2009). Therefore, initially, we inhibited Myo-V using BDM (Uemura et al., 2004) to determine whether this myosin was involved in the targeting of KCa3.1. Data from immunoblot and Ussing chamber experiments demonstrated that BDM reduced the membrane and functional expression of KCa3.1 at the BLM of FRT-KCa3.1-BLAP cells (Figure 6). These data suggest that a Myo-V is required for proper basolateral targeting of KCa3.1.

Several Myo-V isoforms has been reported (Nascimento et al., 2013) and it has been established that the Myo-Vb is implicated in the basolateral trafficking of voltage-gated K^+^ channel Kv1.5 (Schumacher-Bass et al., 2014). Interestingly, Myo-Vc is highly expressed in epithelial (Rodriguez and Cheney, 2002; Jacobs et al., 2009; Nascimento et al., 2013), and this motor protein is known to interact with Rab8 (Jacobs et al., 2009; Xu et al., 2009) which has been reported to play a role in the trafficking of KCa3.1 in epithelial cells (Bertuccio et al., 2014). Endogenous Myo-Vc was knocked down using a siRNA, approach. Based on immunoblot and Ussing chamber experiments conducted on matching passages of cells, a knock down of MyoVc (Figure 7B) was achieved resulting in a corresponding reduced membrane and functional expression of KCa3.1 at the BLM (Figures 7B–D).

Myo-Vc has been investigated in relation to the trafficking pathways the polymeric immunoglobulin receptor (pIgR) and viral interleukin 10 (vIL10) in the immortalized rabbit lacrimal gland acinar cells (LGACs; Xie et al., 2009; Xu et al., 2013). Studies suggest that in LGAC cells the exocytosis of pIgR and vIL10 is dependent on Myo-Vc activity because when Myo-Vc was inhibited there was a reduction in the exocytosis of pIgR and vIL10 in LGACs (Xie et al., 2009; Xu et al., 2013). These data are similar to what we report in this study, suggesting that Myo-Vc is important for targeting of KCa3.1 to the BLM.

However, these findings come with a caveat. While we understand that targeting of KCa3.1 is dependent on Myo-Vc, we are unsure of the exact role Myo-Vc plays. KCa3.1 transport from the Golgi to the BLM is a Rab 8-dependent process (Bertuccio et al., 2014). Rab 8 has been reported to interact with both the actin cytoskeleton and Myo-Vc (Chabrillat et al., 2005; Watanabe et al., 2008; Xu et al., 2009), and as the protein modulates cargo transport between the Golgi and BLM, it is plausible that Myo-Vc also plays a role in cargo transport in this cellular region. However, Myo-Vc has been found to also traffic cargo along microtubules (Jacobs et al., 2009), therefore it is possible that this motor protein transports KCa3.1 along the microtubule cytoskeleton and onto the actin cytoskeleton.

It is also possible that the exocyst may provide this link between the microtubule cytoskeleton and the actin filaments. The exocyst is an octomeric protein complex important for basolateral trafficking in epithelial cells forming a “tether” complex at the BLM (Hsu et al., 2004). Exocyst components Sec6/8 are also involved in the formation of basolateral targeted vesicles (Yeaman et al., 2001). Additionally, the exocyst complex is involved in the targeting and tethering of basolaterally destined vesicles post-Golgi to the membrane, prior to the fusion of the vesicle with the membrane (Munson and Novick, 2006; He and Guo, 2009). Interestingly, we have already demonstrated that the microtubule cytoskeleton is important in the targeting of KCa3.1 and the exocyst complex interacts directly with the yeast Myo-V isoform Myo2p (Reck-Peterson et al., 1999) in the transport of secretory vesicles (Jin et al., 2011). The exocyst also plays a critical role in the development of the microtubule cytoskeleton (Wang et al., 2004) with tubulin polymerization and vesicle targeting to the plasma membrane being reduced in epithelial and neural cells when in exocyst complex is inhibited (Vega and Hsu, 2001; Wang et al., 2004). While this is an unexplored avenue of research it is possible that the exocyst plays an important role in the targeting of KCa3.1, and thus, this warrants further investigation.

Here, we have provided the first evidence that Myo-Vc is critical in the targeting of KCa3.1. Expression and functional studies were employed to investigate both the role of the microtubule and actin cytoskeleton and the motor protein Myo-Vc in the targeting of KCa3.1 to the BLM of polarized epithelial cells. Using pharmacological inhibitors and siRNA techniques it was determined that the basolateral targeting of KCa3.1 in a polarized epithelium is a microtubule, actin microfilament, and Myo-Vc dependent process.

## Author contributions

KH designed the experiments with consultation of RF and ER. RF conducted the experiments. KH, ER, and RF interpreted the experimental results and RF conducted the statistical analyses with advice from KH and ER. All authors approved the final version of the manuscript.

### Conflict of interest statement

The authors declare that the research was conducted in the absence of any commercial or financial relationships that could be construed as a potential conflict of interest.

## Supplementary material

Supplementary Figure 1 **The cytotoxic effects of cytochalasin D (Cyto D), latrunculin A (Lat A), and colchicine on FRT cells. (A)** Cytotoxic effects of Cyto D on confluent FRT-KCa3.1-BLAP cells at 0, 1, 10, and 20 μM were tested over a 5 h time period at incubation intervals of 0, 3, and 5 h at 37°C. Cyto D at 20 μM for *t* = 5 h reduced FRT-KCa3.1-BLAP cell population from 2.1 × 10^6^ ± 3.8 × 10^5^ cells to 1.5 × 10^5^ ± 2.2 × 10^5^ cells (*n* = 4, ^****^*P* < 0.0001). **(B)** Cytotoxic effects of Lat A on confluent FRT-KCa3.1-BLAP cells at 0, 1, 10, and 20 μM tested incubation times of 0, 3, and 5 h at 37°C. Lat A at 20 μM for *t* = 5 h reduced FRT-KCa3.1-BLAP cell counts from 2.8 × 10^6^± 5.2 × 10^5^ cells to 2.0 × 10^6^ ± 9.4 × 10^5^ cells (*n* = 4, ^****^*P* < 0.0001). **(C)** Cytotoxic effects of colchicine at 0, 5, 10, and 20 μM on FRT-KCa3.1-BLAP cells were tested over a 5 h period at incubation intervals of 0, 3, and 5 h at 37°C. Colchicine at 20 μM for *t* = 5 h reduced FRT-cell population from 2.0 × 10^6^ ± 2.0 × 10^5^ cells (*t* = 0 h) to 4.8 × 10^5^ ± 5.2 × 10^4^ cells (*t* = 5 h, *n* = 4, ^****^*P* < 0.0001).

Supplementary Figure 2 **Representative trace of the effects of 1-EBIO, clotrimazole, and barium on K^+^ current of FRT-KCa3.1-BLAP cells**. FRT-KCa3.1-BLAP cells were seeded at a density of 500,000 cells and grown for 72 h on a Snapwell™ insert. Application of 1-EBIO (100 μM) to the FRT-KCa3.1-BLAP cells stimulated a K^+^ current. Once the activated K^+^ current was stable, addition of clotrimazole (10 μM) reduced all of the 1-EBIO-stimulated current. The remaining basal current was decreased to 0 μA with the addition of BaCl_2_ (10 mM; *n* = 2).
