# Supplementary figures and images for: The Role of the Cytoskeleton and Myosin-Vc in the Targeting of KCa3.1 to the Basolateral Membrane of Polarized Epithelial Cells
Source: Front Physiol. 2017 Jan 4;7:639. doi: 10.3389/fphys.2016.00639 (PMC5209343; doi:10.3389/fphys.2016.00639)

Suppl. Figure 1

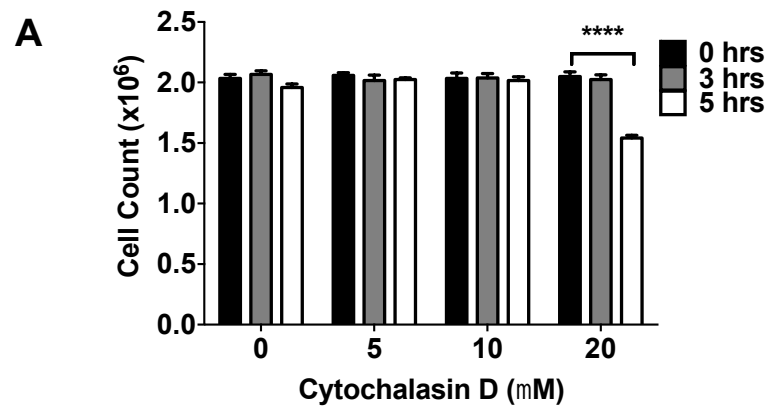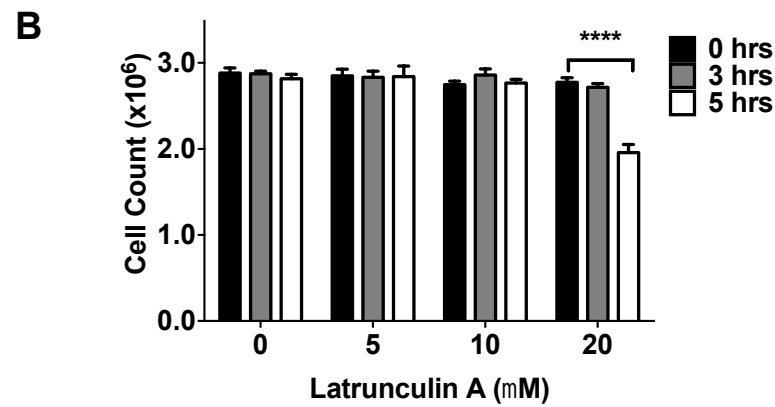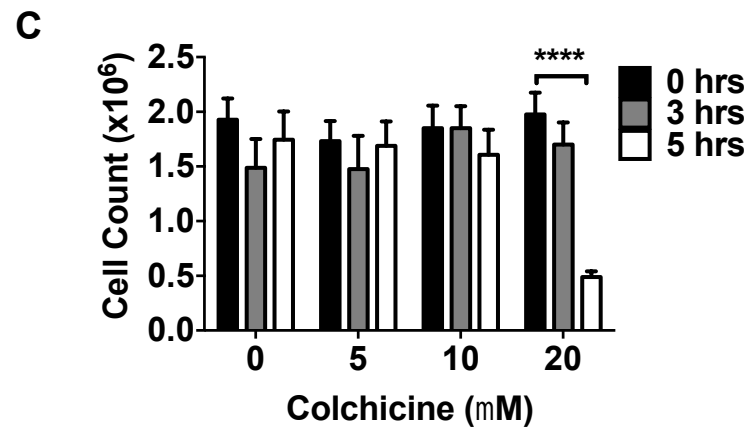

Supplement: Supplementary Figure 1 — The cytotoxic effects of cytochalasin D (Cyto D), latrunculin A (Lat A), and colchicine on FRT cells. (A) Cytotoxic effects of Cyto D on confluent FRT-KCa3.1-BLAP cells at 0, 1, 10, and 20 μM were tested over a 5 h time period at incubation intervals of 0, 3, and 5 h at 37°C. Cyto D at 20 μM for t = 5 h reduced FRT-KCa3.1-BLAP cell population from 2.1 × 10^6^ ± 3.8 × 10^5^ cells to 1.5 × 10^5^ ± 2.2 × 10^5^ cells (n = 4, ****P < 0.0001). (B) Cytotoxic effects of Lat A on confluent FRT-KCa3.1-BLAP cells at 0, 1, 10, and 20 μM tested incubation times of 0, 3, and 5 h at 37°C. Lat A at 20 μM for t = 5 h reduced FRT-KCa3.1-BLAP cell counts from 2.8 × 10^6^± 5.2 × 10^5^ cells to 2.0 × 10^6^ ± 9.4 × 10^5^ cells (n = 4, ****P < 0.0001). (C) Cytotoxic effects of colchicine at 0, 5, 10, and 20 μM on FRT-KCa3.1-BLAP cells were tested over a 5 h period at incubation intervals of 0, 3, and 5 h at 37°C. Colchicine at 20 μM for t = 5 h reduced FRT-cell population from 2.0 × 10^6^ ± 2.0 × 10^5^ cells (t = 0 h) to 4.8 × 10^5^ ± 5.2 × 10^4^ cells (t = 5 h, n = 4, ****P < 0.0001). [file Image1.pdf]

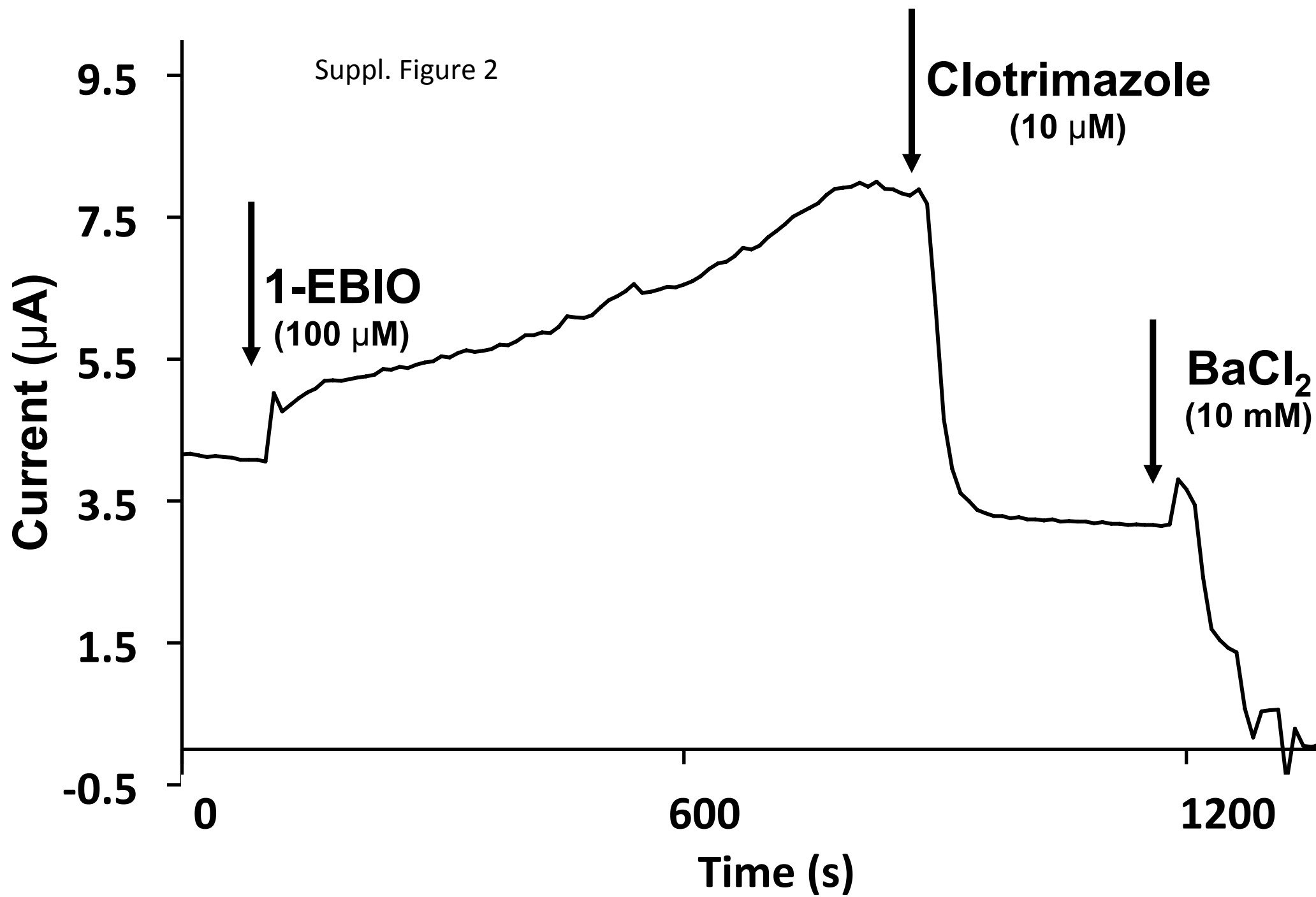

Supplement: Supplementary Figure 2 — Representative trace of the effects of 1-EBIO, clotrimazole, and barium on K+ current of FRT-KCa3.1-BLAP cells. FRT-KCa3.1-BLAP cells were seeded at a density of 500,000 cells and grown for 72 h on a Snapwell™ insert. Application of 1-EBIO (100 μM) to the FRT-KCa3.1-BLAP cells stimulated a K+ current. Once the activated K+ current was stable, addition of clotrimazole (10 μM) reduced all of the 1-EBIO-stimulated current. The remaining basal current was decreased to 0 μA with the addition of BaCl2 (10 mM; n = 2). [file Image2.PDF]
